# Trustworthiness appraisals of faces wearing a surgical mask during the Covid-19 pandemic in Germany: An experimental study

**DOI:** 10.1371/journal.pone.0251393

**Published:** 2021-05-18

**Authors:** Miriam Biermann, Anna Schulze, Franziska Unterseher, Konstantina Atanasova, Paulina Watermann, Annegret Krause-Utz, Dagmar Stahlberg, Martin Bohus, Stefanie Lis

**Affiliations:** 1 Institute of Psychiatric and Psychosomatic Psychotherapy, Central Institute of Mental Health, Medical Faculty Mannheim, University of Heidelberg, Heidelberg, Germany; 2 Institute of Psychology, Leiden University, Leiden, The Netherlands; 3 Chair of Social Psychology, University of Mannheim, Mannheim, Germany; National Institutes of Health, UNITED STATES

## Abstract

**Background:**

During the Covid-19 pandemic, the negative effects of wearing a mouth-nose cover (MNC) on interpersonal functioning have been discussed in public media but empirical studies on how wearing MNCs affect social judgements are sparse. In the present study, we investigated the effects of MNCs on trustworthiness appraisals, the influence of changes due to MNCs in evaluating joy, and the relationship between a social-cognitive appraisal bias and a participant’s characteristics.

**Methods:**

All participants (*N* = 165) judged the intensity of happiness and trustworthiness in calm facial stimuli presented with and without a surgical mask covering part of the face. We analysed the relationship of changes in judgements evoked by MNCs to participants’ evaluations of MNCs as protective tools and explored their associations with the burden experienced by wearing MNCs, compliance to behaviour recommendations, their risk associated with the pandemic, and their levels of psychological distress.

**Results:**

Overall, calm facial stimuli covered with MNCs were evaluated as less trustworthy and, to an even stronger extent, less happy than uncovered facial stimuli. However, participants varied in whether they showed a negative or positive evaluation of faces with MNCs; the negative bias was stronger in those participants who attributed lower protective potential to MNCs, experienced a higher burden while wearing MNCs, wore MNCs less often, and experienced a higher level of psychological distress.

**Conclusions:**

A negative bias in trustworthiness appraisals of faces with a positive emotional expression covered by MNCs is linked to a participant’s evaluation of MNCs as inefficient and burdening and their experience of high psychological distress.

## 1 Introduction

Due to the Covid-19 pandemic, governments across the world currently recommend or even mandate people to wear masks covering the mouth and nose (mouth-nose cover, MNC). MNCs are regarded as an efficient method to limit the distribution of pathogen-carrying aerosols and droplets. Although recent data suggest wearing MNCs can reduce daily growth rates of reported Covid-19 infections by around 45% [1], wearing MNCs is controversial and compliance varies, especially in western cultures. While MNCs have protective properties, wearing them may cause a physical and mental burden [2–4]. MNCs may cause physical stress, particularly for people with pre-existing medical conditions, such as severe obstructive pulmonary disease [e.g. 3]. In addition, they are assumed to increase mental stress during interpersonal encounters; they might make social judgements more difficult by partially hiding facial features indicating another person’s emotions and intentions, thereby hampering smooth social interactions [5–7]. Some authors have already suggested how to deal with these social issues, for example by designing face masks with simple expressive display elements [8] or inducing facial muscle paralysis by botulinum toxin targeting the muscles of the top visible half of the face in order to reduce negative emotions and promote well-being for both the mask-wearer and those who come in contact with that individual [9]. However, empirical studies investigating how MNCs influence the recognition of emotions and complex social judgements are sparse [5, 10, 11]. The present study aims to contribute to a better understanding of interpersonal functioning issues that might arise as a consequence of the widespread use of MNCs during the Covid-19 pandemic in countries where the population is less used to wearing them.

The face is an important source of information that a person uses to identify the individual, recognize the emotional state, and form complex social judgements, such as trustworthiness. Trusting others is a prerequisite for successful social interactions and subjective well-being [12] and influences whether people adapt their beliefs and behaviour to the recommendations of others [13, 14]. Even a stranger’s trustworthiness is assessed within 170 ms of meeting based on facial features [15, 16]. This first impression determines social outcomes, such as electoral success or sentencing decisions [15], although it primarily reflects prejudices instead of an individual’s true trustworthiness [17]. Appraisals of facial trustworthiness are closely related to the evaluation of another person’s affective state: people attribute a higher untrustworthiness to faces with a negative emotional expression such as anger but a higher intensity of trustworthiness to faces with a positive emotional expression, such as joy [e.g. 18, 19].

First studies have revealed that the identification of an individual [6, 7] and the recognition of an emotional state are faultier for faces covered by MNCs [5, 10, 20]. In a sample of 40 healthy participants, recognising basic emotions such as happiness, anger, fearfulness, and disgust was less accurate when faces were covered by MNCs, and participants often misclassified disgusted faces as angry, and joyful faces as neutral, that is, as displaying no emotion at all [5]. These findings are in line with previous studies on face processing; people tend to allocate less time to assessing the eye region but more time to the lower part of a face when determining joy and disgust compared to other emotions [21, 22].

Based on the negative effect of MNCs on the ability to recognise facial emotions, one may expect that MNCs also affect trustworthiness appraisals depending on the specific emotion expressed in a face. Cartaud et al. [10] provided first data on trustworthiness appraisal when a face is covered by an MNC: Masked faces of avatars were assessed as more trustworthy compared to happy, angry and neutral faces without an MNC. However, depending on how a facial emotional expression is misclassified when covered by an MNC, the effects of MNCs may vary. For example, a misclassification of angry faces as disgusted may reduce the intensity of untrustworthiness. In line, the association between a negative, e.g. angry emotional facial expression and untrustworthiness appraisals that can be observed when people judge faces was attenuated when faces were covered by MNCs [11]. In contrast to negative emotional expressions, happy faces covered by MNCs are most often misclassified as neutral expressions [5]. In general, the facial expression of happiness signals the willingness of another to form social bonds [23] and increases an individual’s trustworthiness [19, 24, 25]. Based on the negative effect of MNCs on the ability to assess happiness, one may expect that MNCs also hamper trustworthiness appraisals for faces expressing this positive emotion, thereby exaggerating interpersonal stress during the pandemic. However, although both happiness and trustworthiness judgements overlap in the social-cognitive processes, the eye region of a face is more important for assessing trustworthiness than happiness [18]. This suggests that an MNC might affect trustworthiness appraisals less than joy appraisals, preserving a type of social judgement essential for interpersonal functioning during the pandemic. While an MNC may reduce the wearer’s level of trustworthiness for some people, it might also increase trustworthiness for others. This can be expected when people evaluate MNCs as protective tools. In this case, wearing a mask might signal a person’s intention to protect others from infection and, therefore might even result in the attribution of higher trustworthiness. In line with this assumption, Cartaud et al. [10] found that people assessed faces wearing MNCs as more trustworthy compared to unmasked happy, angry, or neutral facial stimuli (data collected in France at the end of the first quarantine period). However, Cartaud et al. [10] did not characterize the facial expressions of the avatars wearing a mask according to their emotional expression. This hampers the possibility to infer whether this positive bias in trustworthiness through MNCs would also be true for happy faces, even if a face’s trust-promoting features are occluded by an MNC.

In the present study, we investigated the effects of MNCs on trustworthiness appraisals for facial stimuli expressing a low intensity of happiness. Since trustworthiness appraisals are influenced by the emotional state expressed in a face, we compared the appraisal bias evoked by MNCs for trustworthiness judgements to the effects of MNCs on happiness evaluations. Moreover, we analysed whether the changes in judging happiness evoked by MNCs contribute to changes in trustworthiness appraisals. We hypothesised that MNCs would result in a negative bias for trustworthiness, that is, that people will evaluate faces covered with MNCs as less trustworthy compared to fully visible faces, and that effects of MNCs on happiness evaluations will partly predict those on trustworthiness appraisals. However, happiness judgements rely stronger on cues from the mouth region of a face, while trustworthiness ratings are influenced stronger by cues from the eye-region of a face. In line, we expect that covering the mouth-region of a face by an MNC should affect happiness judgments to a higher extent than trustworthiness appraisals.

Finally, we explored which factors are related to the effects of MNCs on social judgements to contribute to understanding interindividual variability during the pandemic. We focused on a person’s evaluation of the benefits and costs related to wearing MNCs and compliance with behaviours suited to reduce the spreading of the Covid-19 infection. In particular, the evaluation of masks as an efficient tool to protect others may be related to higher trustworthiness appraisals of another person’s face with MNCs compared to without [10, 26]. In contrast, a stronger interference of MNCs with social-cognitive judgements might be related to an increased burden during social interactions when people wear MNCs, and a stronger tendency to avoid the use of MNCs. We were also interested in the relationship between judgement biases evoked by MNC with the level of psychological distress and the evaluation of the risk of being infected for oneself and others through the pandemic. Several studies support an increase in psychological distress during the pandemic that affects the general population [27, 28] and, to an even higher extent, patients with a severe mental disorder [29, 30]. Therefore, stress responses have even been reported a considerable time after restrictions such as quarantine and lockdown have been implemented [31]. Furthermore, stress has been linked to a reduced ability to decode emotional facial expressions [32] and an increased sensitivity to positive facial cues, possibly reflecting an attempt to seek social support [33]. To explore whether a high level of distress and a high risk for oneself or others of being infected through the pandemic are related to a stronger bias in social judgements evoked by MNCs, we aimed to include a large proportion of participants with high levels of distress by conducting the study as on online survey posted on the website of the Central Institute of Mental Health (CIMH) in Mannheim, Germany. The CIMH is a psychiatric-psychotherapeutic research institute and university medical centre. It provides information on psychotherapeutic and psychiatric support and treatment, as well as opportunities to participate in different mental health research studies on its website. Therefore, it offers the possibility to reach people who are seeking or exploring possibilities for support during times of mental distress.

## 2 Method

### 2.1 Participants

We recruited 170 participants between 9^th^ July to 27^th^ October 2020, from the website of the CIMH in Mannheim, Germany, and through social networks. During this time period, MNCs were mandatory in Germany on public transport and in shops and services where keeping distance to others was not possible. The study was approved by the Research Ethics Board of the Medical Faculty Mannheim of Heidelberg University. Participants gave their informed consent to start the survey. Of the 170 participants who completed the online survey, we excluded five people from analyses because at the end of the survey they did not confirm to have completed the survey alone and to have answered honestly.

Sociodemographic features: The sample was composed of 86.1% women and 13.3% men (0.6% not specified), with a mean age of 36.04 years (SD 13.63, range 17–71). 85.4% of participants reported being currently employed or in vocational training (7.3% unemployed, 6.1% in retirement, 4.8% not specified). 57.6% were currently in a romantic partnership (38.8% without partner, 3.6% not specified), and 21.2% lived with children (78.8% without children). 53.9% reported to have been in psychiatric treatment at least once in their lives (31.5% in the past, 22.4% currently treated, 43.0% neither in the past nor present, 3.0% not specified).

### 2.2 Measures

Unspecific psychological distress: We measured distress with the Kessler Psychological Distress Scale [K10; 34; German version:, 35]. The K10 yields a global measure of distress based on 10 questions about anxiety and depressive symptoms. The scores range from 10–50, with scores below 20 indicating that a person is likely to be well, and higher scores indicating distress ranging from mild to severe mental disorders. We asked participants to answer the K10 questions for the most recent two weeks. The participants’ mean K10 score of 23.22 (SD 8.52) indicates a mild level of distress: 40.6% of the participants were likely to be well (score 10–19), 21.2% were likely to have a mild disorder (20–24), 13.9% were likely to have a moderate disorder (25–29) and 24.2% were likely to have a severe disorder (30–50).

Covid-19-related risk, the burden related to wearing a face mask and compliance with safety behaviours: To investigate whether changes in social judgements of faces wearing an MNC are linked to cognitions, emotions, and safety behaviour related to the pandemic, we asked participants to answer a set of pandemic-related questions covering four domains. These questions referred to the risk they experienced through the pandemic (‘How do you experience the risk related to the pandemic for a) yourself, b) others, c) your nation, d) the world?’), the protective benefits of face masks (‘How strongly can you protect a) yourself b) others by wearing a face mask?’), the negative effects of wearing a face mask (‘How strong is the a) physical and b) mental burden you experience with wearing a face mask?’), and compliance with behaviours suited to reduce the spreading of the pandemic (‘How often do you wear a face mask?’). All items were answered on a 6-point Likert scale (range 1–6). Responses for the different items were averaged for each of the domains. Means and standard deviations of our sample can be found in S1 Table.

### 2.3 Experimental task and stimulus material

During the experiment, participants judged facial stimuli. We experimentally manipulated the visible part of the face; that is, we presented each face with and without a surgical face mask (independent variable: ‘mask’). Participants assessed how strongly the presented face expressed happiness and trustworthiness (independent variable: ‘task’). They indicated their responses on a 7-point Likert scale ranging from 1–7 (‘not at all’ to ‘very much’).

For facial stimuli, we used calm facial expressions with a straight gaze for 12 different stimulus characters (50% men, 50% women) from the Interdisciplinary Affective Science Laboratory Face Set (IASLab Face Set; IDs of the selected face stimuli: F02, F06, F11, F22, F30, F31, M01, M05, M07, M09, M10, M19). Calm faces have the same valence as happy faces but a lower level of arousal (see affective circumplex model; [36]). Each image was edited with GIMP photo editing software to apply a common surgical face mask, resulting in 24 different stimuli. All faces were presented as greyscale images.

Participants rated each of the 24 stimuli within two separate blocks (happiness vs. trustworthiness). Each block was split into two sub-blocks, which included 12 trials, displaying one of the 12 stimulus characters. Six were presented with masks and six without masks. The trials within each sub-block, the order of the sub-blocks within one block and the type of social judgement were counterbalanced across participants.

### 2.4 Statistical analysis

To analyse whether MNCs influence the intensity to which participants ascribed trustworthiness to a face and whether the change from appraisals of faces without to with MNCs differs between trustworthiness and happiness appraisals, we used a 2x2 rm-ANOVA-design with the repeated measurement factors ‘mask’ (without/with mask) and type of social judgement (‘task’: happiness/trustworthiness). We calculated a linear regression analysis to estimate the extent that variability in the change of trustworthiness appraisals evoked by MNCs is predicted by a change in happiness evaluations. To analyse the relationship between changes in trustworthiness appraisals due to the MNC and participants’ characteristics, we calculated Spearman’s correlation coefficients of these variables with the change in trustworthiness ratings of faces with and without MNCs (with MNC–without MNC, negative scores indicate a negative bias, i.e., a decrease in the intensity of the ascribed trustworthiness). We chose a correlational instead of a regression approach since there is no clear rationale for assigning the changes in social judgements evoked by an MNC and participants’ features to the predictor or outcome variables. We investigated whether a covariation is explained by changes in judging happiness evoked by an MNC by additionally calculating Spearman’s partial correlation coefficients, including changes in the evaluation of happiness related to the MNC as a covariate. To control for multiple testing, we report the corresponding p-values adjusted according to Benjamini and Hochberg [37]. Since the pandemic-related items required the use of non-parametric methods, we chose a more conservative approach for all correlation analyses and consistently report Spearman’s correlation coefficients. Analyses were performed with SPSS 25 or Matlab R2019a.

## 3 Results

### 3.1 Effects of wearing a face mask on social judgements

**Fig 1:**
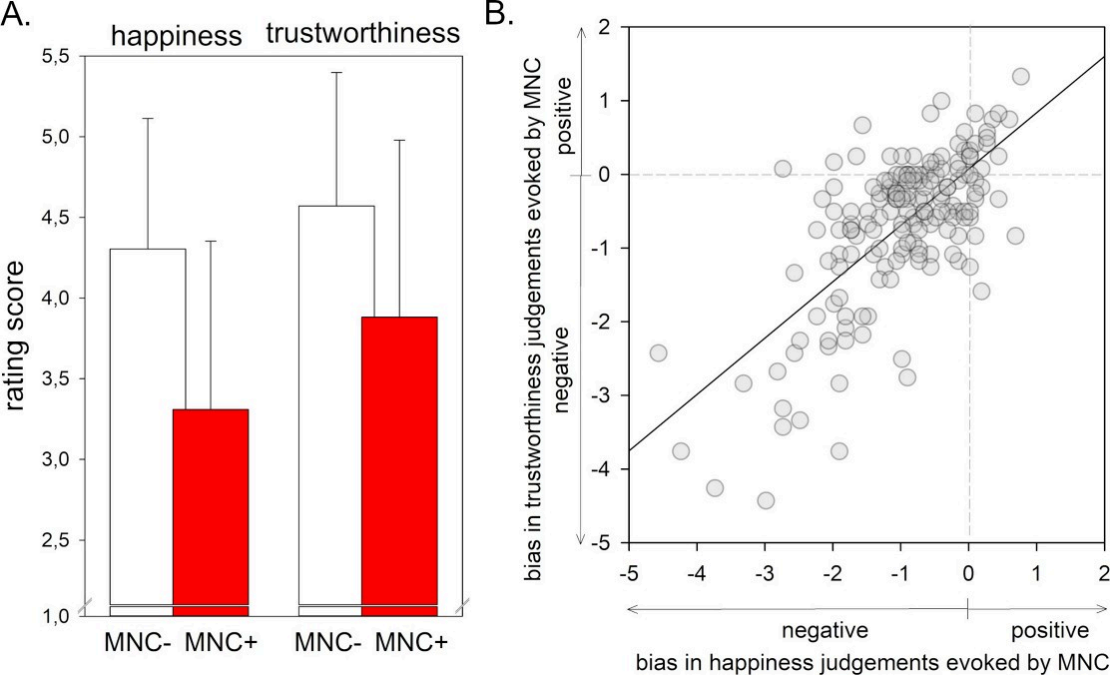
A. Effects of wearing an MNC on the appraisal of happiness and trustworthiness in facial stimuli (*M*, *SD*). B. covariation of the bias in happiness and trustworthiness ratings evoked by MNCs.

Overall, participants rated faces with an MNC as less happy and as less trustworthy than faces without an MNC (main effect ‘mask’: *F*(1, 164) = 140.92, *p* < .001, η_p_² = .462, see Fig 1A). This effect was stronger for happiness evaluations than for trustworthiness ratings (interaction effect ‘type of judgement’ x ‘mask’: *F*(1, 164) = 25.16, *p* < .001, η_p_² = .133, pairwise comparison between levels of ‘mask’ for Happiness: *t* = 13.58, Cohen’s *d =* 1.05 and Trustworthiness: *t* = 8.51, Cohen’s *d* = 0.70). Moreover, trustworthiness ratings were higher than happiness ratings (main effect ‘type of judgement’: *F*(1, 164) = 49.10, *p* < .001, η_p_² *=* .230). Regression analyses revealed that 47% of variance in the change of trustworthiness ratings evoked by an MNC is predicted by the change of happiness ratings (*R*^*2*^ = .48, *b* = .764, *t* = 12.24, *p* < .001, see Fig 1B). However, although overall mean change scores of trustworthiness and happiness ratings were negative, change scores of single subjects revealed also a positive bias, that is, higher happiness and trustworthiness of masked as compared to unmasked faces (see scatterplot in Fig 1B).

### 3.2 Correlation analysis

A stronger negative bias in happiness and trustworthiness appraisals induced by MNCs was linked to a lower attribution of a protective effect to MNCs, a lower experienced risk related to the pandemic, and a higher burden when wearing MNCs (see Table 1). Additionally, for trustworthiness judgements, the negative bias was also stronger in those with a higher level of distress (differences between social judgements in the strength of the correlation coefficients with K10: *z* = 1.79, *p* = .037) and a lower compliance with behaviours suited to reduce a spreading of the pandemic (please note: *r*_s_ did not differ between types of social judgements: *z* = -0.81, *p* = .210). Spearman’s partial correlation coefficients using the bias in happiness appraisals as co-variate confirmed the relations between the strength of a negative bias for trustworthiness judgements with all variables. However, the correlation with the experienced risk of the pandemic and compliance with safety behaviours showed only as a non-significant trend. To explore whether any of the correlations observed for the bias in happiness appraisals is conditionally independent of the trustworthiness bias, we calculated partial correlations with the bias in trustworthiness judgments as co-variate. None of these revealed a significant association (all *p*_*FDR*_ > .370).

**Table 1:** Spearman-Correlation and partial correlation coefficients.

	Change in Happiness (with–without MNC)^a^				Change in trustworthiness (with–without MNC)^a^				Change in trustworthiness with covariate			
									Change in Happiness			
	*r*_*s*_	*p*	*p*_*(FDR)*_		*r*_*s*_	*p*	*p*_*(FDR)*_		*r*_*s*_	*p*	*p*_*(FDR)*_	
Protection MNC	.272	< .001	.005	**	.299	< .001	.003	**	.182	.020	.033	*
Burden MNC	-.249	.001	.003	**	-.321	< .001	.002	**	-.225	.004	.019	*
Compliance	.130	.095	.119		.183	.019	.019	*	.133	.090	.090	(*)
Risk Pandemic	.237	.002	.003	**	.258	.001	.005	**	.153	.050	.062	(*)
K10	-.122	.119	.119		-.239	.002	.003	**	-.208	.008	.019	*

*Note*. ^*a*^ smaller scores indicate a more negative appraisal of faces with an MNC.

*p < 0.05,

**p < 0.01.

To explore whether the negative bias of trustworthiness judgments that is not explained by happiness appraisals was related to specific aspects of the four domains of pandemic-related measures, we performed corresponding analyses for the single items. These partial correlation analyses revealed that the negative bias in trustworthiness appraisals was primarily driven by the risk experienced for oneself (*r*_s_ = .18, *p* = .019; close others: *r*_s_ = .14, *p* = .072, all other *p* > .100), the attribution of a protective function of MNCs (for others: *r*_s_ = .22, *p* = .005; for oneself: *r*_s_ = .09, *p* = .247), mental burden evoked by MNCs (*r*_s_ = -.22, *p* = .004, physical burden: *r*_s_ = .03, *p* = .029) and the compliance with wearing an MNC (*r*_s_ = .17, *p* = .027; avoiding unnecessary activities: *r*_s_ = .13, *p* = .087, all other *p* > .260).

Please note that the pandemic related measures were correlated with each other (for further details see S2 Table).

## 4 Discussion

In the present study, we investigated whether covering a face with an MNC influences complex social judgements such as trustworthiness appraisals for faces with a positive valence. Our data revealed that participants attributed a lower level of trustworthiness to calm faces covered by MNCs compared to faces without any occlusion of facial features. We found this effect also–and to an even stronger extent–for inferring a positive emotional state, such as happiness. These findings are in line with previous studies on emotion recognition in facial stimuli covered by MNCs [5, 10, 11] and with the differential importance of specific facial regions for assessing single emotions in general [18, 21, 22]: The mouth region of the face is particularly important for assessing happiness and disgust, while other emotions and more complex social judgements, such as trustworthiness, rely to a larger extent on the eye region. Moreover, our data support that the evaluation bias in trustworthiness ratings can be partially explained by changes in happiness appraisals evoked by MNCs. This emphasises the importance of the emotional state of the person being judged for trustworthiness judgements, as it has been shown in several studies on first impression trustworthiness appraisals [18, 19, 38].

Beyond the effects of MNCs on social judgements in general, we were interested in whether changes in the appraisal of trustworthiness are related to participant characteristics. Although our data revealed an overall negative bias for trustworthiness ratings of faces wearing MNCs, single subject ratings showed higher trustworthiness appraisals for faces covered by MNCs. In line with our expectations, a participant’s negative bias in trustworthiness appraisals was linked to them attributing lower protective function to MNCs. Additional exploratory analyses suggested that participants who saw MNCs as protecting other people from infection rather than protecting themselves judged faces covered by MNCs as more trustworthy compared to faces without MNCs. Moreover, our exploratory analyses suggested that a negative bias in trustworthiness judgements was also related to a participant’s higher burden caused by wearing MNCs and lower compliance with behaviour recommendations during the pandemic. Finally, we were interested in whether a bias in trustworthiness judgements evoked by MNCs is related to the level of unspecified psychological distress a person has experienced during the pandemic and the risk of being infected for oneself and others they attributed to the pandemic. Our data revealed that people who experienced lower risk associated with the pandemic and a higher level of distress judged faces covered by MNCs as less trustworthy than faces without MNCs. In the same line, people who experienced higher risk and lower distress revealed not only an attenuated bias but some even a positive one. This positive bias is in line with a previous study by Cartaud et al. [10], who showed that avatars wearing MNCs were judged as more trustworthy compared with avatars without MNCs. However, our study suggests that the bias induced by MNCs varies between individuals depending on their attitudes towards the pandemic in general, as well as towards the protective benefits of MNCs in particular. Cartaud et al. [10] provided limited information on characteristics of the participants in their convenience sample, which prevents a direct comparison between findings on the effects of MNCs. Additionally to a positive trustworthiness bias, Cartaud et al. [10] found that participants living in high-risk areas in France during a lock down judged the avatars as less trustworthy and more threatening, independently of whether their faces were covered by MNCs or not. In contrast to these unspecific effects, our findings revealed MNC-related lower trustworthiness appraisals when participants experienced low risk but high levels of distress. To understand these inconsistent findings, it would be essential to investigate whether objective features of an environment such as the level of risk in a geographical area determined by government classifications equals the subjectively experienced risk of an individual and should, in consequence, be linked to comparable changes in social judgments. It seems worth to note that participants of the present study who reported a higher risk also assessed MNCs as a more efficient protective tool and experienced a lower level of distress (see S2 Table). The interplay between these different variables might imply that people differ in the extent to which they experience wearing MNCs as social support within a high-risk environment, that is, as prosocial behaviour aiming to protect others from infection. Experiencing higher social support might result not only in assessing individuals with MNCs as more trustworthy but also attenuate psychological distress [39]. However, an alternative explanation might be that assessing MNCs as protective tool might strengthen self-efficacy in counteracting the subjectively experienced high risk of a pandemic, and thereby reducing distress [40, 41]. Our findings emphasize the need for further studies that take interindividual differences into account when investigating social-cognitive judgments and their link to distress, risk perception, and compliance to behaviours suited to prevent a spreading of infections during a pandemic.

In many aspects, similar relationships as for trustworthiness appraisals can be observed for judging happiness. However, our analyses support that the relationship with changes in trustworthiness cannot solely be explained by a distortion in appraising another person’s emotional state. Our findings point to separate social-cognitive processes involved in the changes to trustworthiness and happiness appraisals evoked by MNCs; the trustworthiness bias was associated with a person’s evaluation of MNCs as protective tools, the burden of wearing MNCs, and the level of unspecified psychological distress, even after taking the bias in happiness judgments into account.

The present study has some limitations. Our findings rely on a convenience sample recruited exclusively from the CIMH website without any restrictions by inclusion or exclusion criteria for participation in the study (except for an age above 18 years). In consequence, our sample is biased in that it includes a high portion of participants who reported to have been in psychiatric treatment at least once in their lives and experience a slightly higher level of unspecific psychological distress compared to convenience samples from different nationalities during the corona pandemic [e.g. 42, 43]. These biases restrict the external validity and the generalisability of our findings. However, our results in this biased sample also allow to disentangle the differential effects of interindividual features, such as experienced distress or risk for changing social judgments towards a higher or lower trustworthiness appraisal. Another feature of our sample that restricts generalisability of the findings is that women were overrepresented in our sample emphasizing the need to replicate our findings in a sample of men. Moreover, the correlations between changes in social judgements and between-subject variability were small, suggesting a shared variance of maximum 10.3%. Since the changes in everyday life during the pandemic vary strongly between people, depending on their personal and social living and working conditions, future studies are required that account for this complexity in the interplay with social-cognitive processing. One example is whether people’s social contacts have been affected by physical distancing: a disruption of social networks during the pandemic attenuated facial happiness appraisals independently of covering a face stimulus with an MNC [20]. However, combining an experimental paradigm with self-report measures always carries the risk of transfer effects. In the present study, self-reports on e.g. distress or attitudes towards MNCs preceded the experimental task. Thereby, it might have functioned as priming influencing subsequent social judgments for faces with and without MNCs. While the frame of reference was similar for all participants, our findings on social judgements should be interpreted with care keeping a possible effect of the context on social judgments in mind.

In sum, our study draws an optimistic picture regarding the consequences of wearing MNCs on social relationships, especially for people who experienced a high risk during the pandemic. The modulating effects of interindividual characteristics suggested that a general negative bias in people making social judgements in mask wearers may not be warranted and may overstate the ubiquity of negativity biases in social-cognitive processes and, in consequence, on social interactions during the pandemic caused by the use of MNCs. This seems to be particularly important, as our study relied on a highly artificial situation with the face as the only source of information on which first-impression social judgements could be based. In everyday life, we combine facial features with a multitude of information provided by different sensory channels, such as change of voice linked to the emotional state, gestures, body postures, or situational context [38]. All of these will strengthen our ability to further improve social judgements when the face is partially covered by MNCs.

## Supporting information

S1 Table Sample description: Pandemic related cognitions, emotions and behaviours. (PDF)

S2 Table Spearman Correlations between pandemic-related measures. (PDF)

S1 Dataset (SAV)
