# Supplementary material for: Trustworthiness appraisals of faces wearing a surgical mask during the Covid-19 pandemic in Germany: An experimental study
Source: PLoS One. 2021 May 18;16(5):e0251393. doi: 10.1371/journal.pone.0251393 (PMC8130962; doi:10.1371/journal.pone.0251393)
Supplement: S1 Table — (PDF) [file pone.0251393.s001.pdf]

## S1 Table

*Sample description: Pandemic related cognitions, emotions and behaviours*

| | Mean | SD* |
|---|---|---|
| Evaluation of the protective potential of MNC | | |
| mean score | 3.59 | 1.23 |
| Self-Protection | 2.81 | 1.45 |
| Protection of others | 4.38 | 1.39 |
| Burden through wearing an MNC | | |
| mean score | 3.08 | 1.57 |
| Emotional burden | 2.93 | 1.85 |
| Physical burden | 3.22 | 1.62 |
| Compliance with safety behaviours | | |
| mean score | 4.42 | 1.00 |
| Wearing a MNC | 5.28 | 1.26 |
| Hand washing | 4.70 | 1.38 |
| Keeping distance of 1.5 m | 4.65 | 1.22 |
| Avoidance of public places | 3.59 | 1.44 |
| Avoiding unnecessary activities | 3.70 | 1.63 |
| Avoiding private travelling | 4.57 | 1.69 |
| Risk assessment | | |
| mean score | 3.83 | 1.10 |
| Risk for oneself | 3.03 | 1.35 |
| Risk for close others | 3.88 | 1.34 |
| Risk for Germany | 3.73 | 1.26 |
| Risk for the World | 4.65 | 1.28 |

*Note.* *standard deviation.
