# Supplementary material for: Trustworthiness appraisals of faces wearing a surgical mask during the Covid-19 pandemic in Germany: An experimental study
Source: PLoS One. 2021 May 18;16(5):e0251393. doi: 10.1371/journal.pone.0251393 (PMC8130962; doi:10.1371/journal.pone.0251393)
Supplement: S2 Table — (PDF) [file pone.0251393.s002.pdf]

S2 Table

Spearman-Correlation coefficients between pandemic related measures

	1	2	3	4	5
1 Protection MNC	-				
2 Burden MNC	-.336**	-			
3 Compliance	.317**	-.297**	-		
4 Risk Pandemic	.529**	-.376**	.566**	-	
5 Psychological Distress	-.102	.359**	-.065	-.133	-

Note. $^{**}p < 0.01$

The evaluation of a protective effect of MNCs was significant positively related to the experienced risk of the pandemic and higher compliance with behaviours suited to reduce the spreading of the pandemic. In contrast, a higher perceived burden through wearing MNCs was related to a lower experienced risk of the pandemic and lower pandemic related compliance behaviours. Higher psychological distress was only related to a higher experienced burden through wearing MNCs.
